# miR-489 inhibits silica-induced pulmonary fibrosis by targeting MyD88 and Smad3 and is negatively regulated by lncRNA CHRF

**DOI:** 10.1038/srep30921

**Published:** 2016-08-10

**Authors:** Qiuyun Wu, Lei Han, Weiwen Yan, Xiaoming Ji, Ruhui Han, Jingjin Yang, Jiali Yuan, Chunhui Ni

**Affiliations:** 1 Department of Occupational Medicine and Environmental Health, Key Laboratory of Modern Toxicology of Ministry of Education, School of Public Health, Nanjing Medical University, Nanjing, China; 2 Institute of Occupational Disease Prevention, Jiangsu Provincial Center for Disease Control and Prevention, Nanjing, China

## Abstract

Silicosis is an incurable occupational disease associated with inflammation, fibroblast proliferation and the accumulation of extracellular matrix in lung tissues. The dysregulation of lncRNAs and miRNAs has been implicated in many complex diseases; however, the current understanding of their roles in fibrotic lung diseases, especially silicosis, remains limited. Our previous microRNA (miRNA, miR) microarray data have indicated decreased expression levels of miR-489 in lung tissues of silica-induced pulmonary fibrosis. Here, we further explored the role of miR-489 in a mouse model of silicosis. Interestingly, miR-489 levels were reduced in both macrophages that were exposed to silica and fibroblasts that were exposed to TGF-β1. Additionally, the overexpressed miR-489 carried out its anti-fibrotic role by attenuating inflammation and fibrotic progression *in vivo*. Our molecular study further demonstrated that miR-489 inhibited silica-induced pulmonary fibrosis primarily by repressing its target genes MyD88 and Smad3. Moreover, the up-regulated lncRNA cardiac hypertrophy-related factor (CHRF) reversed the inhibitory effect of miR-489 on MyD88 and Smad3 and then triggered the inflammation and fibrotic signaling pathways. Overall, our data indicate that the CHRF-miR-489-MyD88 Smad3 signaling axis exerts key functions in silica-induced pulmonary fibrosis and may represent a therapeutic target for silicosis.

Silicosis, caused by the inhalation of crystalline silicon dioxide or silica, is one of the most severe occupational diseases^1^,^2^. Persistent inflammation and pulmonary fibrosis are the most common histological changes^3^. Mounting evidence indicates that silica particles activate macrophages and epithelial cells, causing them to release copious oxidants and cytokines, which in turn results in fibroblast proliferation, epithelial-mesenchymal transition (EMT), deposition of extracellular matrix, and ultimately fibrosis (silicosis)^4^,^5^. Though the pathogenic factor of silicosis is clear, the complex biological and molecular mechanisms underlying silicosis have not yet been fully elucidated.

MicroRNAs (miRNAs, miRs) play a key role in gene regulation by inhibiting the translation of or promoting the degradation of target mRNAs^6^,^7^. An increasing number of studies are screening and identifying miRNAs dysregulated in pulmonary fibrosis. For example, the increase in miR-21 contributes to EMT in pulmonary epithelial cells^8^. miR-199a-5p acts as an effector of TGF-β signaling, regulates Caveolin-1 expression and participates in lung fibroblast activation processes^9^. However, it remains largely unknown how miRNAs regulate pulmonary fibrosis, particularly silicosis. Thus, the identification of specific miRNAs and the clarification of its potential mechanisms mediating silicosis are critical for developing efficient therapies against silicosis.

Long non-coding RNAs (lncRNAs) are non-protein coding transcripts longer than 200 nucleotides. Numerous molecular functions of lncRNAs have been observed, including modulation of proliferation and metastasis^10^,^11^, regulation of cell differentiation^12^, modification of chromatin^13^, and serving as miRNA sponges^14^,^15^. Compared with the well-reported dysregulated miRNAs that participate in diseases, only a small portion of lncRNAs have been investigated regarding their functions in lung cancer, including MALAT1^16^, H19^17^ and HOTAIR^18^, and even fewer have been recognized in pulmonary fibrosis. A recent study has revealed that the long-intergenic non-coding RNAs (lincRNAs) MRAK088388 and MRAK081523 regulate N4bp2 and Plxna4 by acting as ceRNAs that sponge miR-29b-3p and let-7i-5p in bleomycin-induced pulmonary fibrosis^19^. This individual report highlights the urgent need to systematically identify aberrant lncRNAs and to clarify their general mechanism in pulmonary fibrosis.

Our previous miRNA microarray study has shown that miR-489 expression is decreased in lung tissues of silica-induced lung fibrosis^20^. miR-489 has been demonstrated to participate in the maintenance of muscle stem cell quiescence^21^. Moreover, miR-489 appears to play a tumor suppressive role in hypopharyngeal squamous cell carcinoma (HSCC)^22^ and non-small cell lung cancer (NSCLC)^23^. Therefore, miR-489 may be a key molecule in the development of disease. In this study, we observed that miR-489 levels were decreased in a mouse model of silica-induced pulmonary fibrosis. The overexpression of miR-489 blocked pulmonary fibrosis both *in vivo* and *in vitro* by regulating its target genes MyD88 and Smad3, which are critical mediators in the inflammation and fibrotic signaling pathways, respectively. One interesting study has shown that the cardiac hypertrophy-associated lncRNA CHRF acts as an endogenous ‘sponge’ of miR-489, which represses miR-489 activity and functions in cardiac hypertrophy^24^. Here, we hypothesized that the lncRNA CHRF might also downregulate miR-489 expression in silica-induced pulmonary fibrosis. In support of this hypothesis, CHRF has been found to be upregulated in silica-induced pulmonary fibrosis. Further studies have shown that CHRF inhibits miR-489 expression and consequently regulates the target genes MyD88 and Smad3. This study is the first to report that miR-489 attenuates silica-induced pulmonary fibrosis by directly repressing MyD88 and Smad3, and that it is regulated by CHRF. Finally, our study indicates the potential of CHRF and miR-489 to be novel therapeutic targets for silicosis.

## Results

### miR-489 is decreased in mouse lung tissues in a model of silica-induced pulmonary fibrosis

**Figure 1:**
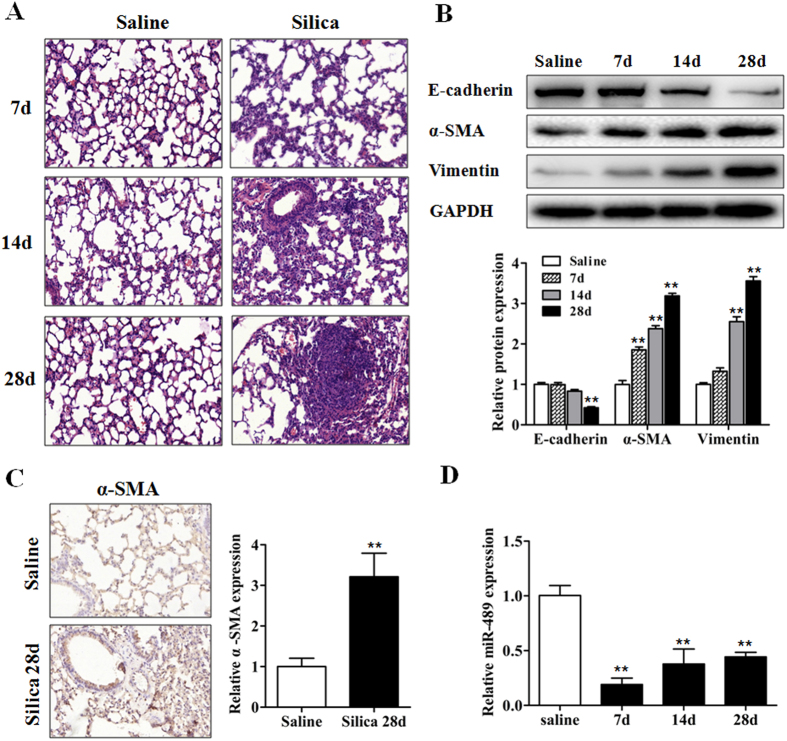
miR-489 is decreased in mouse lung tissues in a model of silica-induced pulmonary fibrosis. **(A)** The C57BL/6 mice were sacrificed on day 7, 14 or 28 after intratracheal instillation of silica suspended saline and saline with *n* = 8 for each group. Histological changes in lung tissues were observed via hematoxylin and eosin (H&E) staining. **(B)** Western blot analysis of the protein expression of E-cadherin, α-SMA and Vimentin in mouse lung tissues from the silica group on day 7, 14 and 28 with **P < 0.01 vs. the day 28 saline group. **(C)** Immunocytochemistry images of the positive-staining areas of α-SMA in mouse lung tissues of the day 28 saline and day 28 silica group with **P < 0.01 vs. the saline group. **(D)** qRT-PCR analysis of miR-489 expression in mouse fibrotic lung tissue on day 7, 14, and 28 (*n* = 8 for each group); U6 was used as an internal control with **P < 0.01 vs. the day 28 saline group. All data are expressed as the means ± SD of at least three independent experiments.

Our previous microarray data have shown that miR-489 levels in mouse lung tissues harvested on day 3, 7, 14, 28 and 56 after the silica injection are lower than that those observed after injection of a saline control (Supplementary Fig. S1)^20^. Interestingly, miR-489 has been reported to be associated with EMT properties in breast cancer^25^. However, the role of miR-489 in silicosis has been unclear. We re-established a mouse model of silica-induced pulmonary fibrosis via intratracheal instillation with silica particles suspended in saline. Histological changes revealed that the structures of the alveolar and air-blood barriers were severely damaged. Inflammatory cell infiltration, diffuse lung fibrosis and fibrotic nodules formation were observed on day 28 after silica instillation (Fig. 1A). These changes were supported by decreased protein levels of epithelial cell marker (E-cadherin) along with increased protein levels of mesenchymal cell markers (α-SMA, Vimentin) (Fig. 1B). Immunohistochemistry staining further showed that the positive-staining areas of α-SMA were significantly higher in mouse lung tissues of the day 28 silica group than those of the day 28 saline group (Fig. 1C). To confirm the change in the miR-489 level after treatment with silica that had been identified in our miRNA microarray data, we examined the expression levels of miR-489 in fibrotic lung tissues by using qRT-PCR analysis and confirmed that the miR-489 levels on days 7, 14, and 28 after silica injection were decreased compared with those in the day 28 saline group (Fig. 1D).

### miR-489 attenuates silica-induced pulmonary fibrosis *in vivo*

**Figure 2:**
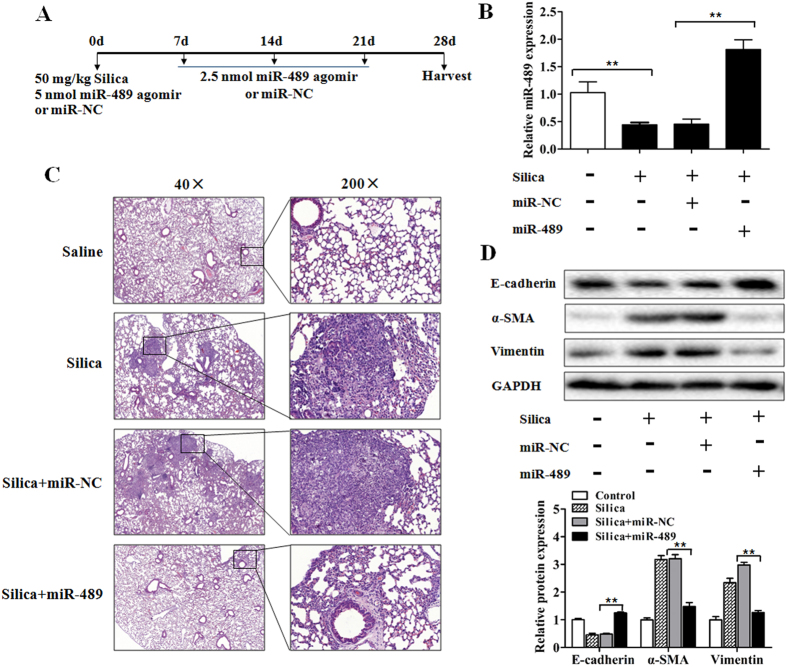
Increased miR-489 attenuates silica-induced pulmonary fibrosis *in vivo*. **(A)** Mouse model of miR-489 overexpression in silica-induced pulmonary fibrosis. The C57BL/6 mice were co-injected with either miR-489 agomir or miR-NC via intratracheal instillation and via the tail vein at the following three time points: day 7, 14 and 21 after silica treatment. Then, lung tissues were harvested on day 28 (*n* = 8 for each group). **(B)** qRT-PCR analysis of miR-489 levels in mouse lung tissues after injection of saline, silica, silica plus miR-NC and silica plus miR-489 agomir for 28 days with **P < 0.01 vs. the saline group or the silica plus miR-NC group. **(C)** The histology of the lung lesions was observed with hematoxylin and eosin (H&E) staining. **(D)** Western blot analysis of E-cadherin, α-SMA and Vimentin expression in saline, silica, silica plus miR-NC and silica plus miR-489 agomir group with **P < 0.01 vs. the silica plus miR-NC group. All data are expressed as the means ± SD of at least three independent experiments.

To explore the potential role of miR-489 in the development of silicosis, silica-induced pulmonary fibrosis mouse models were established as described in the Methods section. To up-regulate miR-489 levels *in vivo*, as shown in Fig. 2A, we co-injected the miR-489 agomir or miR-NC via intratracheal instillation at day 0 and via the tail vein at days 7, 14 and 21 after silica treatment; the lungs were harvested at day 28^26^. As expected, 28 days after silica treatment, miR-489 levels were reduced, whereas miR-489 agomir treatment resulted in increased miR-489 levels compared with those in the silica plus miR-NC group (Fig. 2B). Histological examination via hematoxylin and eosin (H&E) staining showed clear, typical inflammatory and fibrotic nodules in response to silica. However, we observed attenuated inflammation, less severe fibrotic foci (perivascular and peribronchiolar lesions) and less destruction of alveolar architecture in the silica plus miR-489 agomir group (Fig. 2C). Additionally, as shown in Table 1, both the severity and distribution of lung lesions were ameliorated after miR-489 agomir treatment compared with the silica plus miR-NC treatment (*P* < 0.01). Furthermore, the silica-induced elevations in the protein expression of α-SMA and Vimentin were significantly reduced in the presence of miR-489 agomir. Additionally, the protein expression of E-cadherin was restored in the miR-489 agomir treatment group compared with the silica plus miR-NC group (Fig. 2D). Together, these results indicated that the overexpression of miR-489 blocks silica-induced pulmonary fibrosis *in vivo*.

### miR-489 suppresses inflammation by targeting MyD88

**Figure 3:**
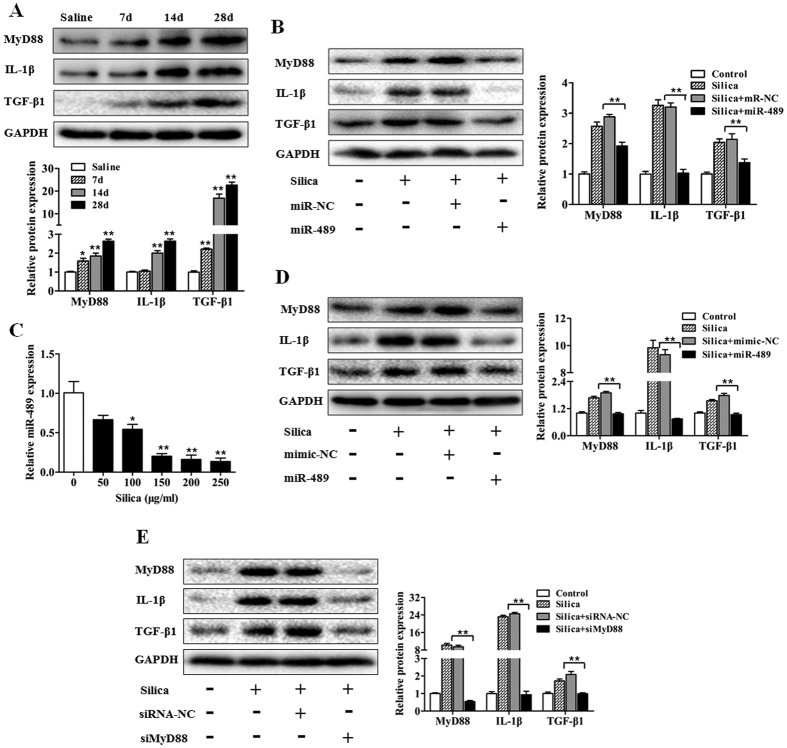
miR-489 suppresses inflammation by targeting MyD88. **(A)** Western blot analysis of the protein expression of MyD88, IL-1β and TGF-β1 in lung tissues on day 7, 14 and 28 after intratracheal instillation of silica-suspended saline with *P < 0.05 and **P < 0.01 vs. the day 28 saline group. **(B)** Western blot analysis of MyD88, IL-1β and TGF-β1 expression in a miR-489 overexpression mouse model with **P < 0.01 vs. the silica plus miR-NC group. **(C)** qRT-PCR analysis of miR-489 levels in RAW 264.7 cells treated with different doses of silica for 12 h with *P < 0.05 and **P < 0.01 vs. the dose 0 group. **(D)** Western blot analysis of MyD88, IL-1β and TGF-β1 expression in RAW 264.7 cells transfected with miR-489 mimic with **P < 0.01 vs. the silica plus mimic-NC group. **(E)** Western blot analysis of MyD88, IL-1β and TGF-β1 expression in RAW 264.7 cells transfected with siRNA against MyD88 for 24 h with **P < 0.01 vs. the silica plus siRNA-NC group. All data are expressed as the means ± SD of at least three independent experiments.

The inflammatory and fibrotic signaling pathways act as important drivers in the development of pulmonary fibrosis. Having demonstrated that miR-489 blocked silica-induced pulmonary fibrosis *in vivo*, we sought to determine how miR-489 suppresses the inflammation induced by silica. To address this question, we attempted to identify potential targets according to their conserved miR-489 binding sites by using bioinformatics databases (microRNA.org, RNAhybrid, miRTarBase). One predicted target of miR-489 is MyD88, which is an important upstream molecule of IL-1β in the inflammatory pathway; moreover, previous studies have reported that miR-489 directly regulates MyD88^24^. In mouse models, we found that MyD88 in fibrotic lung tissue exhibited an increasing trend at different time points after silica treatment (Fig. 3A). In contrast, the overexpression of miR-489 in a mouse model appeared to decrease MyD88 levels (Fig. 3B). These data indicated that miR-489 directly regulates MyD88 expression.

IL-1β is one of the downstream effectors of the MyD88-dependent Toll-like receptors signaling pathway, and fibrogenic factors TGF-β1 are primarily derived from activated and impaired macrophages; we thus hypothesized that miR-489 might affect the IL-1β level by down-regulating the expression of MyD88 and then triggering an inflammatory response, thus further influencing TGF-β1 release. To test this hypothesis, western blot analysis was performed to assess the levels of IL-1β and TGF-β1. IL-1β was significantly increased in the silica group on days 14 and 28, and TGF-β1 was elevated in the silica group on days 7, 14 and 28 (Fig. 3A). The overexpression of miR-489 *in vivo* partially abolished the elevation of IL-1β and TGF-β1 (Fig. 3B). These results suggested that increased expression of miR-489 may indirectly inhibit IL-1β and TGF-β1 levels by regulating MyD88.

To further validate the biological function of miR-489, we treated mouse macrophages (RAW 264.7) with silica and observed that miR-489 levels rapidly decreased (Fig. 3C). As expected, miR-489 overexpression decreased the protein expression levels of MyD88, IL-1β and TGF-β1 (Fig. 3D). To explore the regulatory role of MyD88 in the inflammatory process, siRNA against MyD88 was used to knock down endogenous MyD88 in RAW264.7 cells. As shown in Fig. 3E, the treatment with siRNA against MyD88 resulted in decreased IL-1β and TGF-β1 protein expression. Furthermore, the decrease in miR-489 levels and its target MyD88 were also validated in human differentiated THP-1 macrophages (Supplementary Fig. S2A–C). These data suggest that miR-489 directly targets MyD88 and thereby inhibits the subsequent downstream inflammatory signaling events. Additionally, the MyD88-dependent amplification of an inflammatory cascade indirectly induces the increased synthesis and release of TGF-β1 fibrogenic factors.

### miR-489 suppresses fibroblast differentiation by targeting Smad3

**Figure 4:**
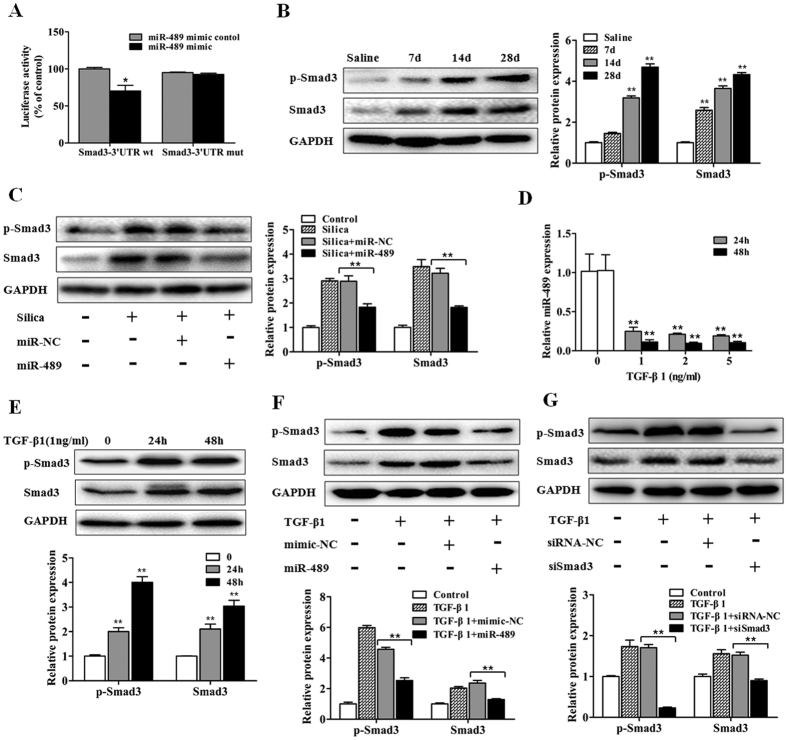
miR-489 suppresses fibroblast differentiation by targeting Smad3. (**A)** Luciferase reporter assays of the relative luciferase activity of NIH/3T3 cells transfected with Smad3-wt and Smad3-mut with *P < 0.05 vs. the miR-489 mimic control group. **(B)** Western blot analysis of total Smad3 and p-Smad3 expression in mouse lung tissues on day 7, 14 and 28 after a single intratracheal instillation of saline or silica with **P < 0.01 vs. the day 28 saline group. **(C)** Western blot analysis of total Smad3 and p-Smad3 expression in a miR-489 overexpression mouse model with **P < 0.01 vs. the silica plus miR-NC group. **(D)** qRT-PCR analysis of miR-489 levels in NIH/3T3 cells treated with different doses of TGF-β1 for 24 and 48 h with **P < 0.01 vs. the dose 0 group. **(E)** Western blot analysis of total Smad3 and p-Smad3 in NIH/3T3 cells treated with 1 ng/ml TGF-β1 for 24 and 48 h with **P < 0.01 vs. the control group. **(F)** Western blot analysis of total Smad3 and p-Smad3 expression in NIH/3T3 cells transfected with miR-489 mimic with **P < 0.01 vs. the TGF-β1 plus mimic-NC group. **(G)** Western blot analysis of total Smad3 and p-Smad3 in NIH/3T3 cells transfected with siRNA against Smad3 for 24 h with **P < 0.01 vs. the TGF-β1 plus siRNA-NC group. All data are expressed as the means ± SD of at least three independent experiments.

Considering the suppression of miR-489 in inflammatory signaling events, we attempted to determine whether miR-489 influences the fibrotic signaling pathways induced by silica. Using bioinformatics databases (microRNA.org, RNAhybrid, TargetScan), we focused on another predicted target of miR-489, Smad3, a key molecule in the TGF-β1 pathway. Therefore, a luciferase reporter assay was used to determine whether Smad3 is a direct target of miR-489. In the presence of miR-489, the relative luciferase activity of Smad3-wt in NIH/3T3 cells was significantly reduced, whereas the Smad3-mut was unaffected (Fig. 4A). These results were in accordance with the results of a previous report^25^ and confirmed that Smad3 is a direct target gene of miR-489.

Next, we assessed whether Smad3 is regulated by miR-489 *in vivo*. We determined the total Smad3 and p-Smad3 levels in fibrotic lung tissue and found that total Smad3 and p-Smad3 expression both increased after silica injection compared with the expression in the day 28 saline group (Fig. 4B). In contrast, down-regulated levels of total Smad3 and p-Smad3 were observed in a miR-489 overexpression mouse model (Fig. 4C). To further explore the regulation of Smad3 by miR-489 in fibroblasts, a mouse fibroblast cell line (NIH/3T3) was treated with various doses of TGF-β1 for 24 and 48 h. miR-489 levels were significantly decreased (Fig. 4D), and the protein levels of total Smad3 and p-Smad3 were increased (Fig. 4E). Moreover, the levels of α-SMA and Vimentin were increased compared with those in the control group (Supplementary Fig. S3A,B), and the overexpression of miR-489 via mimic transfection led to repressed levels of total Smad3 and p-Smad3 (Fig. 4F) and reduced the α-SMA and Vimentin levels in NIH/3T3 cells (Supplementary Fig. S3C).

Next, we determined whether Smad3 is required for the fibrotic process. An siRNA against Smad3 (siSmad3) was used in NIH/3T3 cells. We found that the total Smad3 and p-Smad3 levels were significantly decreased (Fig. 4G) and that the protein expression levels of α-SMA and Vimentin were inhibited by siSmad3 transfection (Supplementary Fig. S3D). Additionally, the decrease in miR-489 levels and the inhibition of miR-489 on its target Smad3 were also confirmed in TGF-β1-treated human lung fibroblasts (MRC-5) (Supplementary Fig. S4A–E). Overall, these data indicated that miR-489 suppresses the fibrotic process by targeting Smad3.

### LncRNA CHRF negatively regulates miR-489 expression

**Figure 5:**
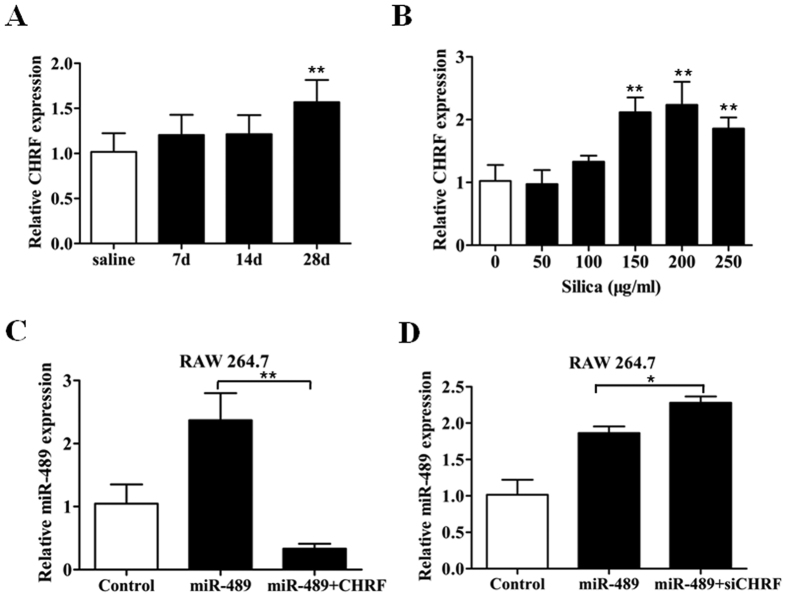
The lncRNA CHRF negatively regulates miR-489 expression. **(A)** qRT-PCR analysis of CHRF levels in mouse fibrotic lung tissue on day 7, 14, 28 (*n* = 8 for each group) with **P < 0.01 vs. the day 28 saline group. **(B)** qRT-PCR analysis of CHRF levels in RAW 264.7 cells treated with different doses of silica for 12 h with **P < 0.01 vs. the dose 0 group. **(C)** qRT-PCR analysis of miR-489 levels in RAW264.7 cells transfected with miR-489 mimic or co-transfected with miR-489 mimic and pUC57 plasmids of CHRF with **P < 0.01 vs. the miR-489 mimic group. **(D)** qRT-PCR analysis of miR-489 levels in RAW264.7 cells transfected with miR-489 mimic or co-transfected with miR-489 mimic and siRNA against CHRF with *P < 0.05 vs. the miR-489 mimic group. All data are expressed as the means ± SD of at least three independent experiments.

Mounting evidence suggests that lncRNAs may participate in competing endogenous RNA (ceRNA) regulatory networks. The lncRNA CHRF has been reported to be an endogenous ‘sponge’ of miR-489 that inhibits the repression of miR-489 on its target genes in cardiac hypertrophy^24^. To investigate whether CHRF plays a role in silica-induced pulmonary fibrosis, we performed qRT-PCR analysis to determine the CHRF levels in mouse fibrotic lung tissues and found that CHRF was significantly increased in the day 28 silica group compared with the day 28 saline group (Fig. 5A). Subsequently, dynamic increases in CHRF levels were also observed in the silica-treated RAW264.7 (Fig. 5B) and TGF-β1-treated NIH/3T3 cells (Supplementary Fig. S5A). These results indicated that CHRF may be crucial in silica-induced pulmonary fibrosis. To explore the possibility that CHRF regulates miR-489 expression, RAW264.7 and NIH/3T3 cells were transfected with miR-489 mimic and then engineered to stably over-express CHRF via CHRF plasmids; knockdown of CHRF was achieved via siCHRF. As expected, the overexpression of CHRF reversed the elevation of miR-489 levels compared with the levels in the miR-489 mimic group in RAW264.7 (Fig. 5C) and NIH/3T3 cells (Supplementary Fig. S5B). In contrast, CHRF knockdown promoted the elevation of miR-489 levels in two cell lines (Fig. 5D, Supplementary Fig. S5C). Thus, these results suggest that CHRF negatively regulates miR-489 expression.

### CHRF promotes silica-induced pulmonary fibrosis through targeting miR-489

**Figure 6:**
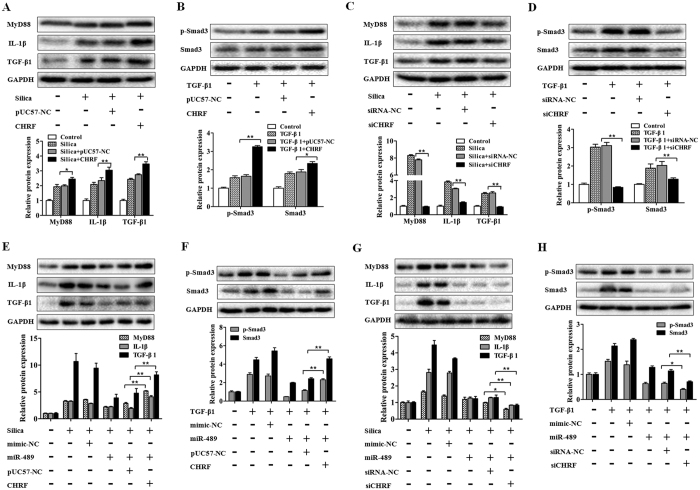
The lncRNA CHRF promotes silica-induced pulmonary fibrosis through targeting miR-489. **(A)** Western blot analysis of MyD88, IL-1β and TGF-β1 expression in RAW 264.7 cells and **(B)** total Smad3 and p-Smad3 expression in NIH/3T3 cells transfected with pUC57 plasmids of CHRF with *P < 0.05 and **P < 0.01 vs. the silica or TGF-β1 plus the pUC57-NC group. **(C)** Western blot analysis of MyD88, IL-1β, TGF-β1 expression in RAW 264.7 cells and **(D)** total Smad3, p-Smad3 expression in NIH/3T3 cells transfected with siRNA against CHRF with **P < 0.01 vs. the silica or TGF-β1 plus the siRNA-NC group. **(E)** Western blot analysis of MyD88, IL-1β and TGF-β1 expression in RAW 264.7 cells and **(F)** total Smad3, p-Smad3 expression in NIH/3T3 cells co-transfected with miR-489 mimic and CHRF plasmids with **P < 0.01 vs. the silica or TGF-β1 plus the miR-489 mimic and pUC57-NC group. **(G)** Western blot analysis of MyD88, IL-1β and TGF-β1 expression in RAW 264.7 cells and **(H)** total Smad3, p-Smad3 expression in NIH/3T3 cells co-transfected with miR-489 mimic and siRNA against CHRF with *P < 0.05 and **P < 0.01 vs. the silica or TGF-β1 plus the miR-489 mimic and siRNA-NC group. All data are expressed as the means ± SD of at least three independent experiments.

We next determined whether CHRF is associated with the progression of silica-induced pulmonary fibrosis. Because miR-489 directly regulates MyD88 and Smad3, the potential effect of CHRF on the expression levels of MyD88 and Smad3 warranted investigation. We found that the overexpression of CHRF induced significant increases in MyD88, TGF-β1, IL-1β (Fig. 6A), Smad3, and p-Smad3 protein levels (Fig. 6B) as well as α-SMA and Vimentin (Supplementary Fig. S6A) levels in RAW264.7 and NIH/3T3 cells. Furthermore, the knockdown of CHRF inhibited increases in MyD88, IL-1β, TGF-β1 (Fig. 6C), Smad3, p-Smad3 (Fig. 6D), α-SMA and Vimentin (Supplementary Fig. S6B) levels induced by silica or TGF-β1 treatment in RAW264.7 or NIH/3T3 cells. Together, our results indicated that CHRF regulates MyD88 and Smad3 and consequently promotes the inflammatory and fibrotic process.

**Figure 7:**
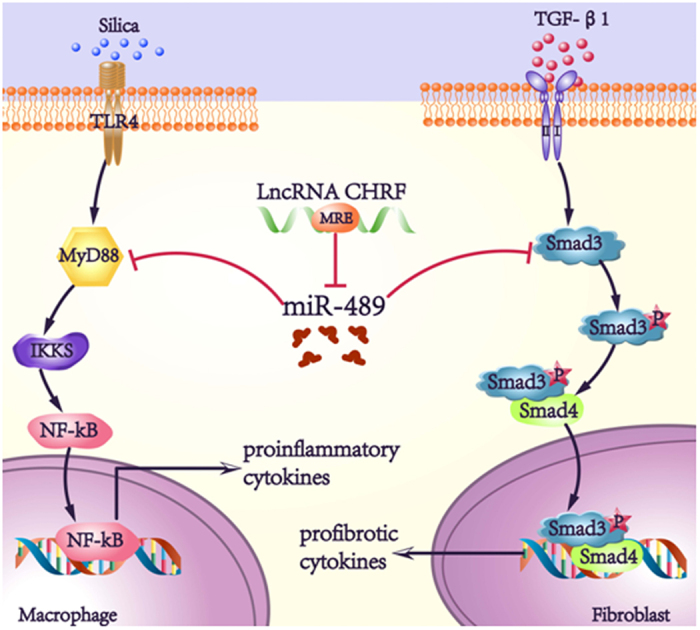
Schematic illustrations explain the signaling mechanisms by which CHRF and miR-489 regulate inflammation and pulmonary fibrosis. MiR-489 targets MyD88 and Smad3, which lead to blockage of the inflammatory and fibrotic signaling pathways in macrophages and fibroblasts. Additionally, the lncRNA CHRF promotes silica-induced inflammation and pulmonary fibrosis by directly down-regulating miR-489 expression.

To confirm that miR-489 was a mediator of CHRF in the pulmonary fibrosis network, the co-transfection methods were used in following experiments. When co-transfecting miR-489 mimic and CHRF plasmid, we observed that the overexpression of CHRF counteracted the repression of miR-489 on its target genes (MyD88, Smad3) (Fig. 6E,F) and promoted inflammatory (IL-1β, TGF-β1) (Fig. 6E) and fibrotic processes (α-SMA, Vimentin) (Supplementary Fig. S6C). Moreover, after co-transfecting miR-489 mimic and siCHRF, we found that the knockdown of CHRF enhanced the roles of miR-489 in inflammatory and fibrotic pathways (Fig. 6G,H, Supplementary Fig. S6D). Most notably, these results strongly suggest that lncRNA CHRF, by targeting miR-489, regulates its targets MyD88 and Smad3 and participates in activating inflammation and pulmonary fibrosis (Fig. 7).

## Discussion

A more complete understanding of miRNAs has prompted researchers to focus on their roles in fibrotic diseases. For example, let-7i negatively regulates angiotensin II-induced cardiac fibrosis by suppressing the expression of interleukin-6 and multiple collagens in the heart^27^. miR-326 regulates expression of TGF-β1 and other profibrotic genes (Ets1, Smad3, and matrix metalloproteinase 9) in idiopathic pulmonary fibrosis (IPF)^28^. miR-29 inhibits the TGF-β1-induced phosphorylation of PI3K-AKT and decreases extracellular matrix synthesis in human lung fibroblasts^29^. Little is known about the roles of miRNAs in the genesis of silicosis, a fibrotic lung disease.

miR-489 has previously been identified as a key regulator of EMT progression, and the overexpression of miR-489 significantly inhibits the migration of Adriamycin (ADM)-resistant human breast cancer cells by blocking EMT signaling^25^. Our present study identified miR-489 as a downregulated molecule in silica-induced pulmonary fibrosis, indicating that altered levels of miR-489 influence the pathogenic mechanism of pulmonary fibrosis. Furthermore, we increased miR-489 levels in both mouse models and cell lines and found that the overexpression of miR-489 attenuated alveolar structural destruction, suppressed inflammation and alleviated silica-induced pulmonary fibrosis. This is the first report demonstrating the functional significance of miR-489 in silica-induced pulmonary fibrosis, and our findings indicate that miR-489 functions as a negative regulator and inhibits the progression of pulmonary fibrosis. Thus, more efforts should be made to develop miR-489 as a novel therapy for pulmonary fibrosis.

Inflammation and the fibrotic process are two primary characteristics of pulmonary fibrosis^30^,^31^; the anti-fibrotic effect of miR-489 may act by regulating these two pathways. miRNAs play important gene-regulatory roles by pairing to the target mRNAs of protein-coding genes and directing their translational repression and/or destabilization^32^. Our present study confirmed that MyD88 is a functional target of miR-489, consistently with the results of previous reports^24^. MyD88 is a key downstream adaptor for most Toll-like receptors and interleukin-1 receptors^33^. The MyD88-dependent signaling pathway activates nuclear factor-kappa B (NF-kB), thereby inducing inflammatory cytokines^34^. One of the crucial families of downstream cytokines of MyD88 is the IL-1 family, whose members possess potent pro-inflammatory properties and have been closely linked to inflammasome proteins^35^,^36^. Consistently with this finding, our studies showed that MyD88 and IL-1β were increased in pulmonary fibrosis and that inverse levels of MyD88 and IL-1β were observed after the overexpression of miR-489. Moreover, the siRNA-mediated knockdown of MyD88 also impaired silica-induced inflammation. These results indicated that the functional mechanism of miR-489 is involved in the regulation of MyD88 and the inflammatory response.

Additionally, when silica particulates enter the alveoli during silicosis, macrophages may be activated and injured. During this scenario, type I alveolar epithelial cells may be injured, and type II alveolar epithelial cells may be activated and differentiated into type I cells, to repair the injured alveoli. In these processes, (with the exception for the release of inflammatory cytokines), macrophages can also derive many fibrogenic factors such as TGF-β1^1^. The key functions of TGF-β1 are to recruit fibroblasts, promote fibroblast proliferation and EMT, which produces large amounts of fibronectin and collagen^37^. An *in-vitro* study has further shown that TGF-β1 induces EMT in bladder cancer cells and increases EMT-associated gene expression^38^. Here we also found that TGF-β1 levels were influenced by miR-489. Therefore, it is likely that miR-489 directly regulates the inflammatory reaction by targeting MyD88 and indirectly induces the increase of TGF-β1 release.

In this study, we further demonstrated that Smad3 is another target of miR-489; this finding is supported by previous reports^25^. Smad3 is one of the most important regulatory proteins in the TGF-β1/Smad3 signaling pathways. Smad3 deficiency abolishes the TGF-β1-induced expression of α-SMA and extracellular matrix proteins and inhibits the EMT pathway^39^,^40^. Here, Smad3 was found to be relatively highly expressed in a mouse fibrosis model, and cell lines, and this expression was decreased by miR-489 overexpression. Moreover, fibroblasts transfected with siRNA against Smad3 experienced opposite effects (as predicted), which resulted in the attenuation of silica-induced fibrosis. This finding provided further evidence of miR-489 as an inhibitor that targets Smad3 and then blocks the subsequent downstream signaling events of the silica-induced fibrotic process. Previous results have shown that miR-489 may target many other genes in addition to the MyD88 and Smad3 targeted in this study. Thus, the identification of these target genes and discussions of their signaling pathways may shed new light on the function of miR-489 in silica-induced pulmonary fibrosis. Additionally, we chose to study only macrophages and fibroblasts because both have been confirmed to be the major cell types responsible for silica-induced lung fibrosis *in vivo*. It is unknown which cell type or types had decreased miR-489 expression in the present study. This issue will be explored in our future study.

LncRNAs have recently been reported to act as miRNA sponges or miRNA inhibitors (antagomirs), which interact with miRNAs and modulate the expression of miRNA target genes^41^,^42^. For example, lncRNA UCA1 acts as a ceRNA, which effectively down-regulates miR-216b, thereby modulating the derepression of FGFR1 and activating the ERK signaling pathway in hepatocellular carcinoma^43^. The lncRNA HULC acts as an endogenous ‘sponge’, which down-regulates miR-372 and reduces the repression of its target gene, PRKACB^44^. Recent reports have indicated that a lncRNA (CHRF) directly binds to miR-489 and regulates MyD88 expression in cardiac hypertrophy^24^; we investigated whether CHRF has similar effects on miR-489 levels in silica-induced pulmonary fibrosis. CHRF was significantly increased in both a mouse model and cell lines, and an inhibitory effect of CHRF on miR-489 levels was further observed in macrophages and fibroblasts. Moreover, the overexpression of CHRF aggravated inflammation and fibrosis, whereas the knockdown of CHRF inhibited inflammation and fibrosis. These data indicated that CHRF may directly or indirectly participate in inflammation and fibrosis. We further confirmed that the overexpression of CHRF reversed the inhibitory effects of miR-489 on its target genes MyD88 and Smad3. The knockdown of CHRF promoted the functions of miR-489. These results revealed that CHRF is involved in silica-induced pulmonary fibrosis through regulating both the expression and the function of miR-489. However, the exact mechanism by which CHRF regulates miR-489 remains unknown and warrants investigation.

However, there are some limitations to the present study: first, the regulation of miR-489 by CHRF has not yet been validated in humans because the human and mouse sequences have limited homology; second, other factors that may contribute to lung inflammation and fibrosis have not been explored. Therefore, further mechanism studies are needed to determine the relationships and regulatory interactions among CHRF, miR-489 and key molecules.

In conclusion, as shown in Fig. 7, miR-489 may function as an anti-fibrotic miRNA in silica-induced pulmonary fibrosis by targeting MyD88 and Smad3, and the lncRNA CHRF inhibits miR-489 expression, thus resulting in the activation of inflammation and the fibrotic signaling pathways. Therefore, the current findings not only provide new insight in clarifying the complex molecular mechanisms of specific miRNAs and lncRNAs in silica-induced pulmonary fibrosis but also facilitate the development of miRNA and lncRNA-directed therapeutic strategies for this disease.

## Materials and Methods

### Ethics statement

All animal procedures were conducted in accordance with humane animal care standards, and all experimental protocol were approved by the Nanjing Medical University Ethics Committee (Nanjing, China).

### Animals

For the mouse model of pulmonary fibrosis, C57BL/6 male mice (4–6 weeks of age) were purchased from the Shanghai Laboratory Animal Center (SLAC, Shanghai, China). A total of 48 C57BL/6 mice were randomly divided into 6 groups (*n* = 8 in each group): day 7, 14, and 28 saline groups (control) and day 7, 14, and 28 silica groups. The mice were anesthetized using pentobarbital sodium (Dainippon Sumitomo Pharma, Osaka, Japan) through intraperitoneal injection. The mice were instilled with 50 mg/kg of silica (Sigma Aldrich, USA) in 0.05 ml sterile saline or 0.05 ml sterile saline intratracheally. The mice were sacrificed on day 7, 14 or 28 after instillation, and the lungs were harvested and stored at −80 °C immediately for further analysis.

### miR-489 overexpression mouse model of pulmonary fibrosis

A total of 32 C57BL/6 male mice were randomly divided into 4 groups (*n* = 8 in each group): saline, silica, silica plus miR-NC and silica plus miR-489 agomir. The methods of anesthesia, saline and silica treatment were the same as mentioned above. Either 5 nmol of miR-489 agomir or miR-NC (RiboBio Co., Ltd., Guangzhou, China) was co-administered with silica-suspended saline. Subsequently, 2.5 nmol of miR-489 agomir or miR-NC was injected via the tail vein into each mouse weekly. The mice were sacrificed on day 28 after silica administration, and the lungs were isolated and stored at −80 °C.

### Cell culture and treatment

The mouse macrophages (RAW 264.7) and fibroblasts (NIH/3T3), as well as the human monocytes (THP-1) and fibroblasts (MRC-5) were purchased from the American Type Culture Collection (ATCC, Manassas, VA, USA). All cell lines were maintained in Dulbecco’s modified Eagle’s medium (DMEM, Life Technologies/Gibco, Grand Island, NY) or RPMI Medium 1640 basic (1640, Life Technologies/Gibco, Grand Island, NY) containing 10% fetal calf serum (FCS, Life Technologies/Gibco, Grand Island, NY), 100 U/ml penicillin and 100 μg/ml streptomycin (Life Technologies/Gibco, Gaithersburg, MD). The cell lines were cultured in a humidified atmosphere containing 5% CO_2_ at 37 °C. The human macrophages were acquired by using THP-1 cells differentiated with 100 nM PMA (Phorbol-12-myristate-13- acetate, Sigma, St. Louis, MO, USA) for 48–72 h.

For western blot or qRT-PCR analysis, macrophages and fibroblasts were plated (2 × 10^5^ cells) into 6-well plates overnight. The macrophages were treated with 100 μg/ml silica for 12 h. The fibroblasts were treated with 1 ng/ml TGF-β1 (Sigma-Aldrich) for 48 h, and the total RNA or protein was prepared according to the experimental instructions.

### Histology and immunohistochemistry

All lung tissues from each treated mouse were subjected to histological examination and hematoxylin and eosin staining. For hematoxylin and eosin staining, mouse lung tissues were fixed for 48 hours using 4% paraformaldehyde and subsequently embedded in paraffin overnight and then sectioned at 6 μm thicknesses and stained using hematoxylin and eosin according to the manufacturers’ instructions.

### Immunohistology

The blank slices of mouse lung tissues underwent blocking of endogenous peroxidase activity by incubation in 3% H_2_O_2_ for 10 min and microwave antigen retrieval followed by incubation with anti-α-SMA antibody (1:200, Abcam, Cambridge, UK) at 4 °C overnight. Specimens were incubated for 30 min with secondary antibody, then diaminobenzidine (DAB) solution was used. Counterstaining was performed with hematoxylin. The 3D HISTECH panoramic viewer was used for imaging histologic sections and immunohistological sections.

The degrees of severity and distribution were used to determine lesion injury including alveolar wall thickening, inflammatory lesions, collagen expression and fibrosis. A grading system was utilized for each group of animals, as described in a previous report. For lesion severity: 0 = nothing/zero, 1 = marginal, 2 = slight, 3 = moderate, 4 = severe and 5 = very severe. For lesion distribution: 0 = absent, 1 = rare/occasional (10% of the lung area), 2 = sparse/limited (10–25% of the lung area), 3 = moderate (25–50% of the lung area), 4 = extensive/widespread (50–75% of the lung area) and 5 = very extensive/predominant (over 75% of the lung area)^45^.

### RNA isolation and quantitative real-time PCR

Total RNA from cultured cells and mouse tissues was extracted using Trizol (Life Technologies/Ambion, Carlsbad, CA, USA) according to the manufacturer’s protocol. The concentration and quality of the RNA were confirmed using a Thermo NanoDrop 2000 spectrophotometer. To quantify CHRF, 500 ng of total RNA was reverse-transcribed using random primers in a 10-μl reaction. To measure miR-489, 500 ng of total RNA was reverse-transcribed using specific primers in a 10-μl reaction. Reverse transcription was performed as described using the PrimeScript^TM^ RT reagent Kit (TaKaRa Bio Inc, Japan). The SYBR^®^ Premix Ex Taq^TM^ II kit (TaKaRa Bio Inc, Japan) was used for CHRF and miR-489 amplification. The primers for CHRF and the bulge-loop™ miRNA qRT-PCR Primer Sets (one RT primer and a pair of qPCR primers for each set) specific for miR-489 were designed by RiboBio Co., Ltd. (Guangzhou, China). The sequences of CHRF primers were forward: 5′-GTGTTAGCCACCACTACCCAGC-3′; reverse: 5′-CCACAGCCCACAACTTTCA AG-3′. qRT-PCR analysis was performed using an ABI 7900HT Real-Time PCR System (Applied Biosystems). miR-489 expression was normalized to U6. CHRF expression was normalized to that of GAPDH. The relative expression levels were analyzed using the 2^−ΔΔCT^ method.

### Western blotting

The proteins in lung tissues and cultured cells were extracted in lysis buffer (M-PER reagent for the cells and T-PER reagent for the tissues, Thermo Scientific). A total of 80 μg protein extracts was electrophoresed on 12.5% polyacrylamide gradient gels and then transferred to nitrocellulose membranes. The membranes were incubated in blocking buffer (5% milk) prior to incubation with primary antibodies at 4 °C overnight. After incubation with the corresponding secondary antibody (Beyotime Bio, China), membrane analysis was performed using a ChemiDoc XRS+ imaging system (Bio-Rad Laboratories, Inc.).

Antibodies specific to Smad3 (ab40854, 1:2,000), p-Smad3 (ab52903, 1:2,000), α-SMA (ab124964, 1:2,000) and IL-1β (ab9722, 1:2,000) were purchased from Abcam. Antibodies specific to MyD88 (D80F5, 1:500), Vimentin (D21H3, 1:1,000), E-cadherin (24E10, 1:1,000) and GAPDH (13E5, 1:1,000) were obtained from Cell Signaling Technology. Anti-TGF-β1 (sc-146, 1:200) was obtained from Santa Cruz.

### Luciferase assays

The wild type 3′UTR sequence of Smad3, which contains miR-489 binding sites (Smad3 3′UTR-wt), and its mutant sequence (Smad3 3′UTR-mut) were chemically synthesized (RiboBio Co., Ltd., Guangzhou, China) and inserted into the psiCHECK-2 plasmid (Promega). Approximately 1 × 10^4^ NIH/3T3 cells per well were seeded into 96-well plates in triplicate, then 0.4 μg of smad3 3′UTR wt or mut psiCHECK-2 plasmid was co-transfected with 20 nM miR-489 mimic (miR-489) or mimic control (mimic-NC) into NIH/3T3 cells using transfection reagent (RiboBio Co., Ltd., Guangzhou, China) according to the manufacturer’s instructions. Firefly and Renilla luciferase activities in cell lysates were determined using the dual-luciferase assay system (Promega) according to the manufacturer’s protocol and normalized as the quotient of Renilla/firefly luciferase activities.

### miRNA and siRNA transfection

Approximately 1 × 10^4^ macrophages or fibroblasts were seeded in 6-well plates containing 2 ml of DMEM or 1640 for 24 h. Subsequently, 25 nM of miR-489 or mimic-NC (RiboBio Co., Ltd., Guangzhou, China) was incubated with cells in each well for another 24 h, and this was followed by silica or TGF-β1 treatment. Cells were harvested for assays at 12 hours after silica treatment or 48 hours after TGF-β1 treatment.

MyD88 siRNA (siMyD88) and Smad3 siRNA (siSmad3) (RiboBio Co., Ltd., Guangzhou, China) were diluted as suggested by the protocol. Non-targeting siRNA (siRNA-NC) was used as the negative control. SiMyD88 or siSmad3 and siRNA-NC were transfected into macrophages or fibroblasts and then treated with silica or TGF-β1 according to the previously described methods.

### Immunofluorescence

NIH/3T3 cells were plated onto coverslips and placed in a laser scanning confocal vessel. After TGF-β1 treatment, the cells were fixed with 4% (m/v) carbinol, washed with 0.1% PBST, and blocked with 5% BSA. They were then incubated with primary antibody overnight at 4 °C. The primary antibody was specific for α-SMA (ab124964, 1:200, Abcam). Finally, the cells were stained with anti-rabbit secondary antibody and DAPI in the dark, after being washed with 0.1% PBST. A 3D HISTECH panoramic viewer was used for cell imaging.

### Plasmids construction and cell transfection

The CHRF lncRNA sequence was synthesized and subcloned into pUC57 to generate CHRF plasmids (GenScript, Nanjing, China). The overexpression of CHRF was achieved via transfection with CHRF plasmids into RAW 264.7 and NIH/3T3 cells. An empty pUC57 (pUC57-NC) was used as the control. Small interfering RNAs targeting CHRF (siCHRF) were designed and synthesized by RiboBio Co., Ltd. Non-targeting siRNA (siRNA-NC) was used as a negative control. For CHRF plasmid transfection, 1 × 10^5^ cells were cultured on a 6-well plate, transfected with 5 μg/well CHRF plasmids or pUC57-NC for 24 h, and subjected to silica or TGF-β1 treatment. For siCHRF transfection, the cells were seeded into 6-well plates, transfected with 150 nM/well siCHRF or siRNA-NC for 24 h, then subjected to silica or TGF-β1 treatment. The transfection reagent (RiboBio Co., Ltd., Guangzhou, China) was used according to the manufacturer’s instructions.

### Statistical Analysis

All data are expressed as the means ± SD of at least three independent experiments. Data were analyzed using independent-samples *t* tests between two groups and one-way analysis of variance (ANOVA) for more groups with Dunnett’s test (dose 0 as the control group). A value of *p* < 0.05 was considered significant.

## Additional Information

**How to cite this article**: Wu, Q. *et al*. miR-489 inhibits silica-induced pulmonary fibrosis by targeting MyD88 and Smad3 and is negatively regulated by lncRNA CHRF. *Sci. Rep.* **6**, 30921; doi: 10.1038/srep30921 (2016).

## Supplementary Material

Supplementary Information

## Figures and Tables

**Table 1:** Effect of miR-489 agomir administration on lung histopathology.

Groups	Lesion severity grade						Average severity grade	Lesion distribution grade						Average distribution grade
	0	1	2	3	4	5		0	1	2	3	4	5	
Saline	8						—	8						—
Silica		2	1	1	3	1	3.00 ± 1.51		1		6	1		2.88 ± 0.83
Silica+miR-NC		2	2		1	3	3.13 ± 1.81		2	2	3	1		2.38 ± 1.06
Silica+miR-489	2	2	4				1.25 ± 0.89	2	6					0.75 ± 0.46

Values represent the means ± SD of 8 for each group, **P < 0.01 vs. silica plus miR-NC group (independent-samples *t* test).
