# Supplementary material for: miR-489 inhibits silica-induced pulmonary fibrosis by targeting MyD88 and Smad3 and is negatively regulated by lncRNA CHRF
Source: Sci Rep. 2016 Aug 10;6:30921. doi: 10.1038/srep30921 (PMC4978961; doi:10.1038/srep30921)
Supplement: Supplementary Information [file srep30921-s1.pdf]

miR-489 inhibits silica-induced pulmonary fibrosis by targeting MyD88 and Smad3 and is negatively regulated by lncRNA CHRF

Qiuyun Wu[1,†], Lei Han[1,2, †], Weiwen Yan[1,†], Xiaoming Ji[1], Ruhui Han[1], Jingjin Yang[1], Jiali Yuan[1], Chunhui Ni[1,*]

[1] Department of Occupational Medicine and Environmental Health, Key Laboratory of Modern Toxicology of Ministry of Education, School of Public Health, Nanjing Medical University, Nanjing, China.

[2] Institute of Occupational Disease Prevention, Jiangsu Provincial Center for Disease Control and Prevention, Nanjing, China.

[†]These authors contributed equally to this work.

[*] Corresponding authors

[*] Correspondence to: Department of Occupational Medicine and Environmental Health, Key Laboratory of Modern Toxicology of Ministry of Education, School of Public Health, Nanjing Medical University, Nanjing 211166, China. Tel: +86 25 86868418; Fax: +86 25 86868499; Email: chni@njmu.edu.cn, chninjmu@126.com

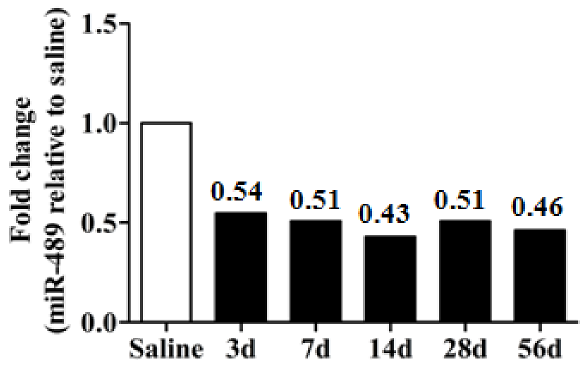

Figure S1. miR-489 is decreased in mouse lung tissues in a model of silica-induced pulmonary fibrosis. The C57BL/6 mice were sacrificed on day 3, 7, 14, 28 or 56 after intratracheal instillation of silica suspended saline and saline. The fold changes of miR-489 in mouse lung tissues were determined by microRNA (miRNA, miR) microarray with total RNA analysis.

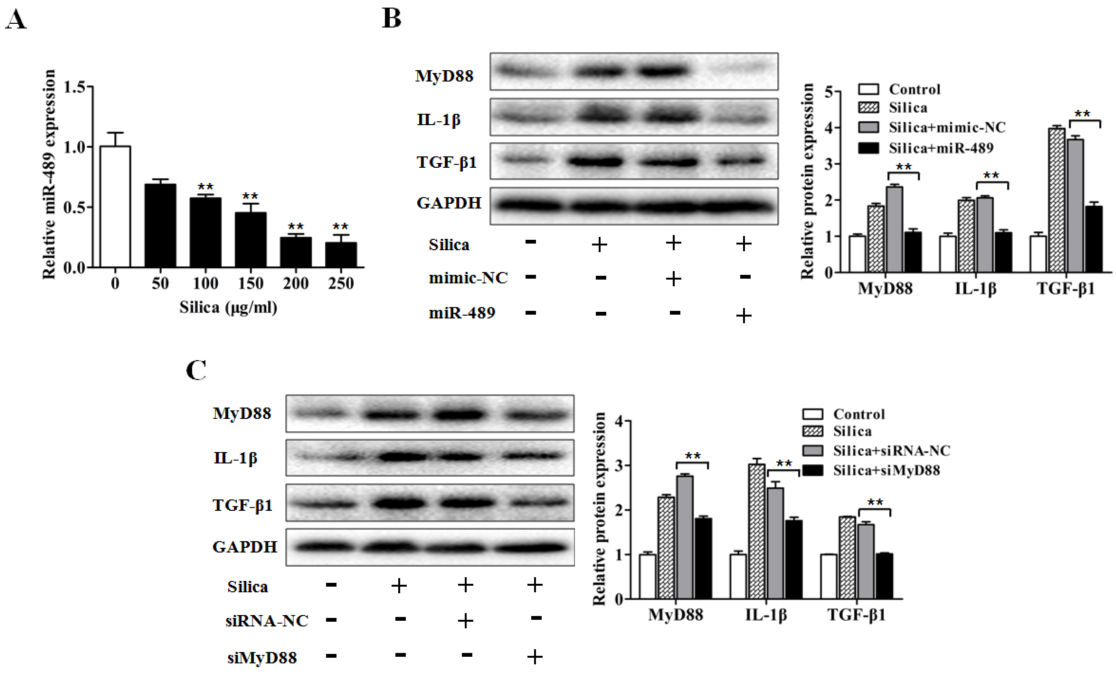

Figure S2. miR-489 suppresses inflammation by targeting MyD88 in differentiated THP-1 cells. (A) qRT-PCR analysis of miR-489 levels in differentiated THP-1 cells treated with different doses of silica for 12 h with ***P*<0.01 vs. the dose 0 group. **(B)** Western blot analysis of MyD88, IL-1β and TGF-β1 expression in differentiated THP-1 cells transfected with miR-489 mimic with ***P*<0.01 vs. the silica plus mimic-NC group. **(C)** Western blot analysis of MyD88, IL-1β and TGF-β1 expression in differentiated THP-1 cells transfected with siRNA against MyD88 for 24 h with ***P*<0.01 vs. the silica plus siRNA-NC group. All data are expressed as the means ± SD of at least three independent experiments.

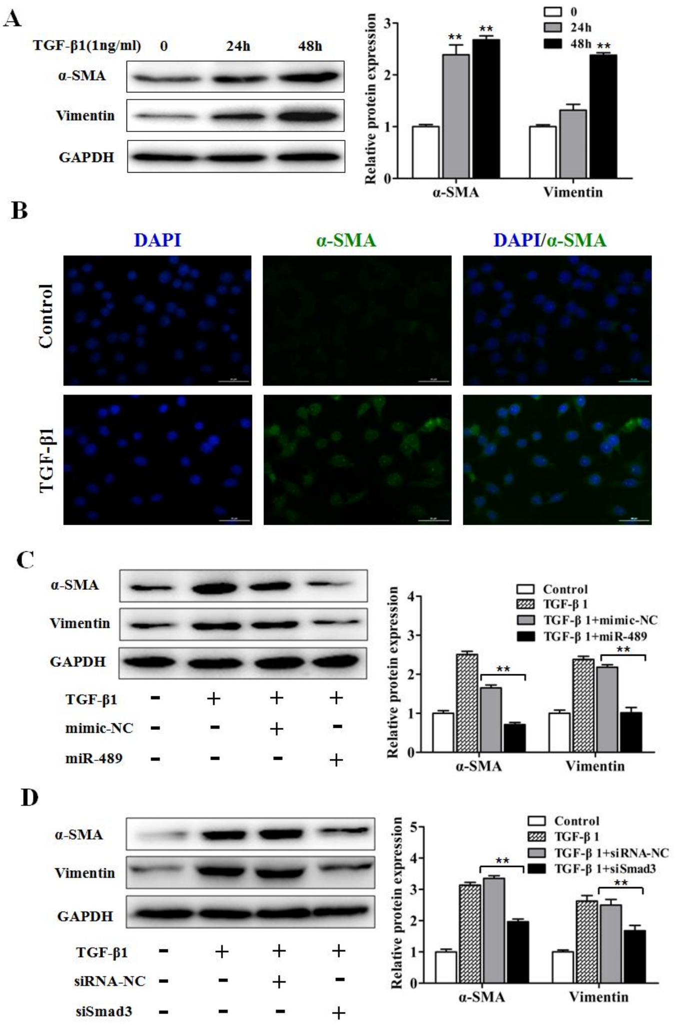

Figure S3. miR-489 suppresses fibrotic biomarkers by targeting Smad3. (A) Western blot analysis of α-SMA and Vimentin expression in NIH/3T3 cells treated with 1 ng/ml TGF-β1 for 24 and 48 h with **$P<0.01$ vs. the control group. **(B)** Immunofluorescence images of α-SMA expression in NIH/3T3 cells treated with 1 ng/ml TGF-β1 for 48 h. **(C)** Western blot analysis of α-SMA and Vimentin expression in NIH/3T3 cells transfected with miR-489 mimic with **$P<0.01$ vs. the TGF-β1 plus mimic-NC group. **(D)** Western blot analysis of α-SMA and Vimentin expression in NIH/3T3 cells transfected with siRNA against Smad3 for 24 h with **$P<0.01$ vs. the TGF-β1 plus siRNA-NC group. All data are expressed as the means ± SD of at least three independent experiments.

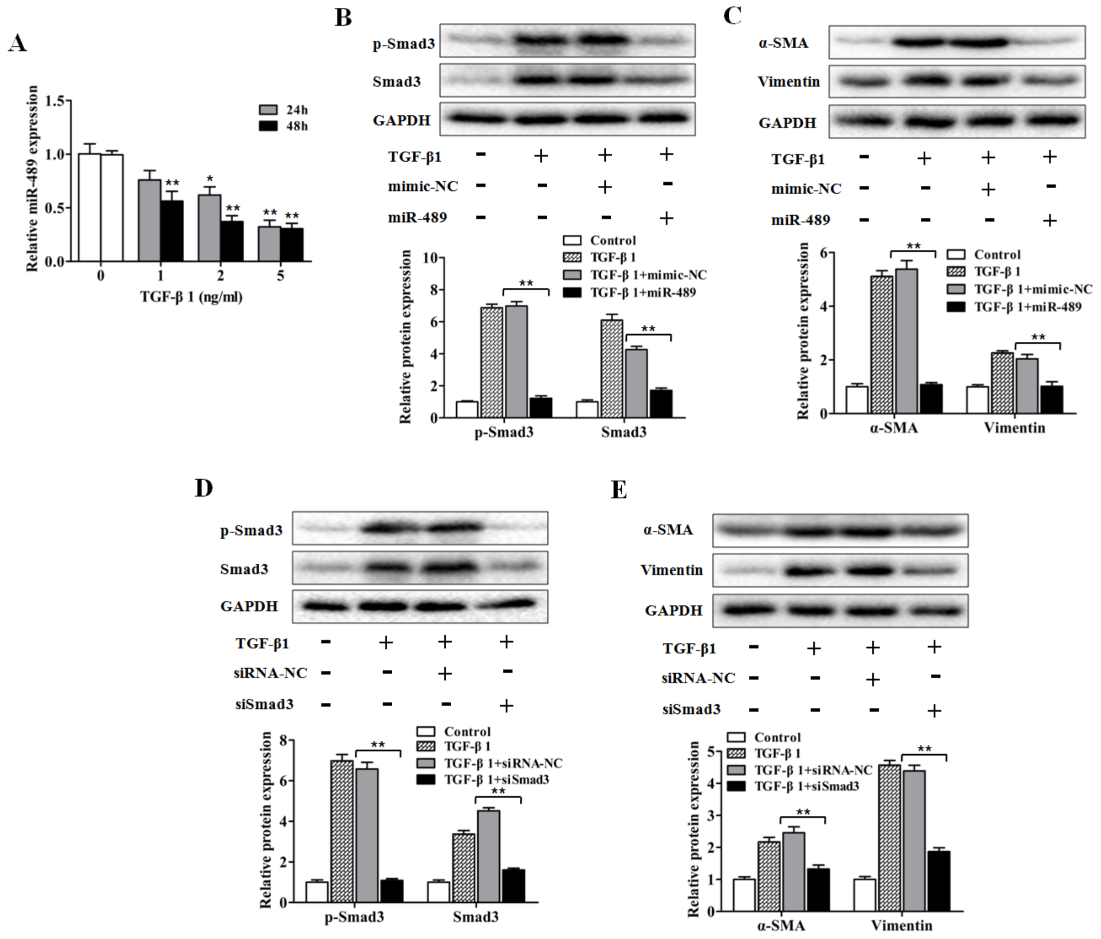

Figure S4. miR-489 suppresses fibroblast differentiation by targeting Smad3 in human fibroblasts (MRC-5). (A) qRT-PCR analysis of miR-489 levels in human fibroblasts (MRC-5) treated with different doses of TGF-β1 for 24 and 48 h with **P*<0.05 and ***P*<0.01 vs. the dose 0 group. **(B-C)** Western blot analysis of total Smad3, p-Smad3, α-SMA and Vimentin expression in MRC-5 cells transfected with miR-489 mimic with ***P*<0.01 vs. the TGF-β1 plus mimic-NC group. **(D-E)** Western blot analysis of total Smad3, p-Smad3, α-SMA and Vimentin expression in MRC-5 cells transfected with siRNA against Smad3 for 24 h with ***P*<0.01 vs. the TGF-β1 plus siRNA-NC group. All data are expressed as the means ± SD of at least three independent experiments.

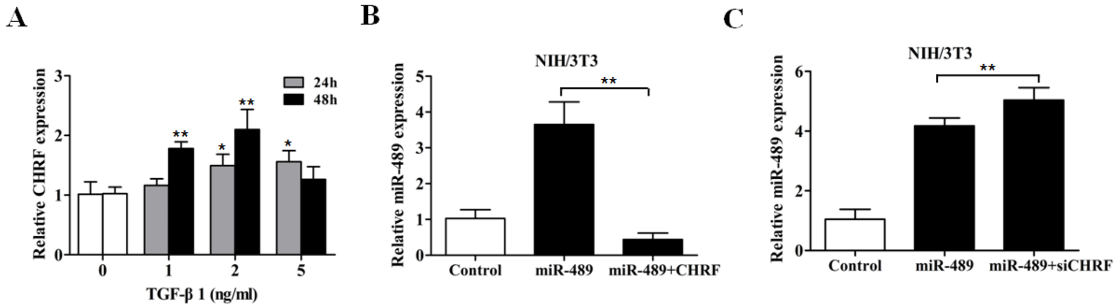

Figure S5. The lncRNA CHRF negatively regulates miR-489 expression in fibroblasts. (A) qRT-PCR analysis of CHRF levels in NIH/3T3 cells treated with different doses of TGF-β1 for 24 and 48 h with *$P<0.05$ and **$P<0.01$ vs. the dose 0 group. **(B)** qRT-PCR analysis of miR-489 levels in NIH/3T3 cells transfected with miR-489 mimic or co-transfected with miR-489 mimic and pUC57 plasmids of CHRF with **$P<0.01$ vs. the miR-489 mimic group. **(C)** qRT-PCR analysis of miR-489 levels in NIH/3T3 cells transfected with miR-489 mimic or co-transfected with miR-489 mimic and siRNA against CHRF with **$P<0.01$ vs. the miR-489 mimic group. All data are expressed as the means ± SD of at least three independent experiments.

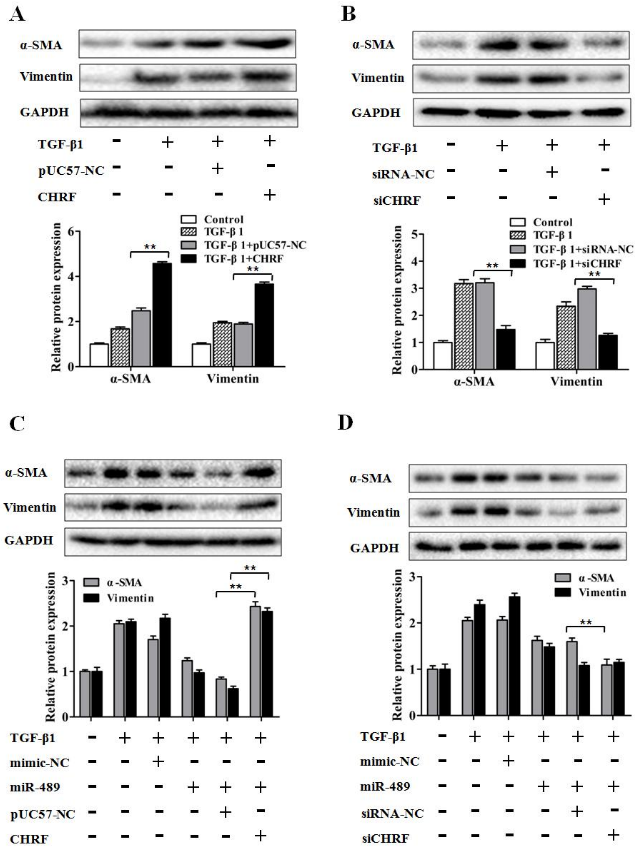

Figure S6. The lncRNA CHRF promotes silica-induced elevation of fibrotic biomarkers through targeting miR-489. (A) Western blot analysis of α-SMA and Vimentin expression in NIH/3T3 cells transfected with pUC57 plasmids of CHRF with ***P*〈0.01 vs. the TGF-β1 plus pUC57-NC group. **(B)** Western blot analysis of α-SMA and Vimentin expression in NIH/3T3 cells transfected with siRNA against CHRF with ***P*〈0.01 vs. the TGF-β1 plus siRNA-NC group. **(C)** Western blot analysis of α-SMA and Vimentin expression in NIH/3T3 cells co-transfected with miR-489 mimic and CHRF plasmids with ***P*〈0.01 vs. the TGF-β1 plus miR-489 mimic and pUC57-NC group. **(D)** Western blot analysis of α-SMA and Vimentin expression in NIH/3T3 cells co-transfected with miR-489 mimic and siRNA against CHRF with ***P*〈0.01 vs. the TGF-β1 plus miR-489 mimic and siRNA-NC group. All data are expressed as the means ± SD of at least three independent experiments.
